# Lithium-Renal and brain induced toxicity

**DOI:** 10.1192/j.eurpsy.2022.1832

**Published:** 2022-09-01

**Authors:** M. Zrelli, E. Bergaoui, N. Staali, K. Souabni, W. Melki

**Affiliations:** 1 Razi Hospital, Psychiatry D, Manouba, Tunisia; 2 Razi Hospital, Psychiatry D, Tunis, Tunisia

**Keywords:** Polypharmacy-lithium toxicity, renal toxicity, neurotoxic effect

## Abstract

**Introduction:**

Lithium can induce renal and neurotoxic effects, particularly if it is combined with a neuroleptic or if there is an intercurrent condition. The neurological sequelae may be irreversible.

**Objectives:**

To show the renal and neurotoxic effects of lithium and the risk of its association with haloperidol.

**Methods:**

A case of irreversible lithium neurotoxicity wih renal sequelae.

**Results:**

This case report is about a 57-year-old patient with a bipolar disorder type 1. She was well stabilized on lithium.On December 2020, the patient had an increased level of creatinine, therefore her medication was stopped. She developed a manic episode then she was switched on Haldol 25mg and lithium. After 4 days, she had a neuroleptic malignant syndrome with renal and neurological sequelae.. She was referred to us after her discharge from intensive care. The patient was agitated, anxious, sad with a superficial contact and a well-structured speech. She had delusional ideas of prejudice about her husband. On physical examination, she had a parkinsonian syndrome, moderate organic renal failure with a clearance of 45.93 ml/min. On the cerebral MRI, she had a diffuse cotico-subcortical atrophy with bilateral frontal predominance and vascular leukopathy. The most probable cause was the iatrogenic effects of the association of lithium and haloperidol. We decided to stop all medications and the patient got better.

**Conclusions:**

Recognizing the neurotoxic effect of lithium and making an early diagnosis is a crucial determinant in the evolution of the disease and its irreversibility. Polypharmacy and comorbidities appear to be important precipitating factors for lithium toxicity.

**Disclosure:**

No significant relationships.

